# “Inhibition of mitochondrial carbonic anhydrases prevents endothelial cell death and blood brain barrier dysfunction in models of amyloidosis.”

**DOI:** 10.1002/alz.093285

**Published:** 2025-01-09

**Authors:** Nicole L Lemon, Rafael Vazquez‐Torres, Elisa Canepa, Rebecca M Parodi Rullan, Marc A Ilies, Silvia Fossati

**Affiliations:** ^1^ Alzheimer’s Center at Lewis Katz School of Medicine, Temple University, Philadelphia, PA USA; ^2^ Temple University School of Pharmacy, Philadelphia, PA USA

## Abstract

**Background:**

The Neurovascular Unit is a multicellular structure of the CNS known to become dysfunctional in Alzheimer’s Disease (AD) and cerebral amyloid angiopathy. Amyloidosis disrupts the function of cerebrovascular endothelial cells (cECs) via extrinsic and intrinsic apoptosis, and induction of blood brain barrier (BBB) permeability. Findings in our lab demonstrated that pan‐Carbonic Anhydrase inhibitors (CAi’s) prevent mitochondria‐mediated apoptotic mechanisms in cECs. Therefore, we hypothesized that the mitochondrial CA isoforms, CA‐VA and ‐VB mediate Aβ‐induced cell death and other deleterious mechanisms in cECs. Importantly, mitochondrial isoform CA‐VB is increased in models of amyloidosis. Thus, we anticipate using a pharmacological selective CA‐Vi will reduce cerebrovascular EC apoptosis and prevent EC activation. We also hypothesize that CA‐VB KO in human cerebral cECs will rescue mitochondrial‐mediated apoptosis and protect from BBB dysfunction. Finally, we anticipated that CA‐Vi prevents cognitive decline/AD pathology in the 3xTG mouse model.

**Method:**

Human cerebral microvascular ECs (hCMECs) were treated with Aβ40‐Q22 Dutch mutant alone, and in combination with CA‐Vi, and we measured outcomes of mitochondria‐mediated apoptosis and BBB function. In parallel to pharmacological inhibition, we employed CA‐VB KO hCMECs to understand the contribution of CA‐VB to mechanisms of EC apoptosis and BBB function. To confirm the findings *in vivo*, we treated 3xTG AD mice with CA‐Vi from 6 to16 months of age. Mice went through behavior paradigms, and tissue was harvested. We then measured BBB and cell death outcomes, in addition to Aβ and tau levels.

**Result:**

Inhibition of CA‐V prevented Aβ40‐Q22‐induced mitochondria‐mediated EC death and activation. CA‐VB KO in hCMECs also protected against Aβ‐induced cell death and BBB permeability. Importantly, CA‐Vi prevented cognitive decline, Aβ and tau accumulation, and EC activation in 3xTG mice.

**Conclusion:**

Inhibition of mitochondrial CA‐V is protective in models of amyloidosis and AD in vitro and in vivo through the prevention of mitochondria‐mediated cell death and loss of BBB function. Future studies will aim to understand the cell‐specific mechanisms of CA‐V inhibition.

